# Replacing a Swiss ball for an exercise bench causes variable changes in trunk muscle activity during upper limb strength exercises

**DOI:** 10.1186/1476-5918-4-6

**Published:** 2005-06-03

**Authors:** Gregory J Lehman, Trish Gordon, Jo Langley, Patricia Pemrose, Sara Tregaskis

**Affiliations:** 1 Department of Graduate Studies, Canadian Memorial Chiropractic College, Toronto, ON, Canada; 2 Undergraduate Department, Canadian Memorial Chiropractic College, Toronto, ON, Canada

**Keywords:** EMG, exercise, spine stability, swiss balls, rehabilitation, low back pain

## Abstract

**Background:**

The addition of Swiss balls to conventional exercise programs has recently been adopted. Swiss balls are an unstable surface which may result in an increased need for force output from trunk muscles to provide adequate spinal stability or balance. The aim of the study was to determine whether the addition of a Swiss ball to upper body strength exercises results in consistent increases in trunk muscle activation levels.

**Methods:**

The myoelectric activity of four trunk muscles was quantified during the performance of upper body resistance exercises while seated on both a stable (exercise bench) and labile (swiss ball) surface. Participants performed the supine chest press, shoulder press, lateral raise, biceps curl and overhead triceps extension. A repeated measures ANOVA with post-hoc Tukey test was used to determine the influence of seated surface type on muscle activity for each muscle.

**Results & Discussion:**

There was no statistically significant (p < .05) difference in muscle activity between surface conditions. However, there was large degree of variability across subjects suggesting that some individuals respond differently to surface stability. These findings suggest that the incorporation of swiss balls instead of an exercise bench into upper body strength training regimes may not be justified based only on the belief that an increase spinal stabilizing musculature activity is inherent. Biomechanically justified ground based exercises have been researched and should form the basis for spinal stability training as preventative and therapeutic exercise training regimes.

**Conclusion:**

Selected trunk muscle activity during certain upper limb strength training exercises is not consistently influenced by the replacement of an exercise bench with a swiss ball.

## Background

The use of physioballs/Swiss balls in strength and conditioning programs has become ubiquitous. Swiss balls have been incorporated into strength training regimes and touted as a means to more effectively train the musculoskeletal system. Performing strength exercises on Swiss balls has been advocated on the belief that a labile surface will provide a greater challenge to the trunk musculature, increase the dynamic balance of the user and possibly train users to stabilize their spines to prevent and treat injury.

Despite a few studies, the research supporting these ideas is sparse. Vera-Garcia et al [1] documented increases in rectus abdominis and external oblique activity during curl ups when performed on a Swiss ball compared with a stable surface. Mori [2] documented trunk muscle activation levels during a variety of trunk muscle exercises showing that substantial levels of trunk muscle activity occurs. However, because the exercise tasks were not also performed on a stable surface it is unknown how much the Swiss ball's instability contributed to a demand for muscle activation. Andersen [3] investigated the influence of a Swiss ball on upper limb muscle activation and force production during a chest press. The study found that while muscle activation in the primary movers was not different between surfaces, the amount of force generated was significantly less on a Swiss ball. These results were mirrored in a previous study [4] investigating force and muscle activation of the lower extremity on unstable surfaces.

Swiss balls are currently used to replace stable benches during the performance of upper body strength training exercises. While previous work has documented the myoelectric activity of the trunk muscles during exercises specifically designed to train the trunk muscles, no study has documented the effect of an unstable surface on trunk muscle activity during resistance exercises for the upper limbs. Due to common use of Swiss balls this lack of research is significant for both performance and safety concerns (i.e. Swiss balls may increase the risk of falling without providing an exercise benefit). Adequate spinal stability is important in the prevention and treatment of low back injuries [5]. Stability is achieved through the coactivation of trunk muscles; therefore, endurance training has been postulated to be beneficial in training trunk muscles to provide stability. It is possible that performing upper body strength exercises on a swiss ball can increase trunk muscle activity to a sufficient extent to adequately stress the spinal stabilizing musculature to achieve beneficial endurance training effects. This may render conventional trunk resistance exercises superfluous and increase the efficiency of rehabilitation and prophylactic exercise programs. Contrarily, an elevated muscle activation level may be contraindicated in subjects with low back injury or unstable spines. Co-activation of the trunk muscles has a compressive loading cost that may outweigh the benefits of trunk muscle training. Safe exercises on stable ground have been advocated and thoroughly investigated with a detailed biomechanical model [6] which provide an excellent balance between muscle stress and low compressive/shear penalty, the same can not be argued for the majority of exercises incorporating the use of Swiss balls.

In light of the popularity of Swiss balls and the lack of research investigating their influence on trunk muscle activity during upper limb strength exercises, it was the aim of this study to determine if the use of a Swiss ball instead of an exercise bench results in consistent increases across subjects in trunk muscle activation levels during upper body strength training exercises.

## Methods

### Participant Characteristics

Seven healthy males (average age (standard deviation) 28 (3.8), average height in cm (standard deviation) 179.7 (7.13) and average mass in kg 84.6 (8.09) and five females (age = 23.6 kg(.8), height 168.3 cm(5.04) and mass 61 kg(5.2)) with weight lifting and abdominal exercise experience, were recruited from a convenience sample consisting of college students. Subjects currently experiencing low back pain or a history of low back pain within 3 months were excluded from the study. Participants read and signed an information and consent form approved by the institutions Internal Review Board.

### Experimental Design

A single factor repeated measures design was used to analyze the effect of trunk muscle activity during common weight training exercises on a Swiss ball compared to the trunk muscle activity found during the performance of the exercises on an exercise bench. All subjects performed 6 different exercises on two different support surfaces for a total of 12 separate movement tasks during a single testing session. The order of the tasks was kept constant with every subject.

#### Instrumentation

EMG data was collected using disposable bipolar Ag-AgCl disc surface electrodes with a diameter of one cm, adhered bilaterally over the muscle groups with a centre-to-centre spacing of 2 cm. Raw EMG was amplified between 1000 and 20,000 times, depending on the subject. The amplifier had a CMRR of 10,000:1 (Bortec EMG, Calgary AB, Canada). Raw EMG was band pass filtered (10 and 1000 HZ) and A/D converted at 2000 Hz using a National Instruments data acquisition system.

### Electrode Placement

Skin preparation included shaving (when necessary) and cleansing and abrading the skin with alcohol solution prior to applying the electrodes to reduce skin impedance. Four sites on participants' right sides were chosen for electrode placement: (1) rectus abdominus (RA) 3 cm lateral to the umbilicus, (2) External Oblique (EO) 15 cm lateral to the umbilicus oriented in the direction of the muscle fibres (3) internal oblique (IO) 10 cm lateral to midline (inferior (2 cm) to the ASIS angled superomedial to inferolateral parallel to the underlying muscle fibers and (4) erector spinae 2 cm lateral to L4–L5 interspinous space in a superomedial to an inferolateral orientation over the muscle fibers. A reference electrode was placed over the eleventh rib. The investigators may have made slight modifications and adjustments for any anatomical variations between subjects.

### Normalization task procedure

Subjects were required to perform maximum voluntary contractions for the trunk musculature. Subjects were required to perform a 3 second maximal supine isometric trunk curl up and bilateral twist against an immovable resistance to maximally recruit the rectus abdominis, external oblique and internal oblique. Subjects, performed this movement supine with the spine in neutral. Second, the subjects performed an isometric prone trunk extension against a fixed resistance to maximally recruit the lumbar erector spinae. All subjects performed the normalization tasks in the same order. Participants practiced the exercises before the collection of data. The muscle activity during all subsequent exercise tasks was expressed as a percent of the peak activity found during the normalization procedure. The peak activity was found visually after the signal had been processed in an identical manner to the exercise tasks.

### Exercises Performed

Subjects performed six exercise tasks. The six exercises were modified to be performed while seated on the labile surface of an exercise ball resulting in a total of 12 separate movement tasks:

1.a) Supine abdominal curls on a flat bench with feet flat on floor and folded across chest.

1.b) Modified; supine abdominal curl on a Swiss ball with feet flat on floor and Swiss ball positioned under the low back.

2.a) Supine dumbell chest press on a flat bench, with feet flat on the floor. Subjects started with weight at chest level and hands shoulder width apart. Subjects pressed weight up until elbows were extended.

2.b) Modified; supine chest press on a Swiss ball with feet flat on the floor and Swiss ball positioned under the shoulders and thoracic spine.

3.a) Seated shoulder press on flat bench with no back support and feet on the floor. Subjects started with weights at shoulder level and pressed up to full elbow extension.

3.b) Modified; seated shoulder press on Swiss ball.

4.a) Seated lateral shoulder raise on flat bench. The weighted straight arm was abducted to 90 degrees from 0 degrees of abduction.

4.b) Modified; seated lateral shoulder raise on a Swiss ball.

5.a) Seated two arm biceps curl on flat bench. Subjects started in the anatomical position and flexed the arms bilaterally.

5.b) Seated two arm biceps curl on Swiss ball.

6.a) Seated double arm overhead triceps extension on a flat bench. Subjects began with their shoulders and elbows fully flexed and the weight behind their head. The elbow was then extended to raise and lower the weight.

6.b) Modified; seated double arm overhead triceps extension on a Swiss ball.

Exercises 2–6 were performed with dumbbells. Each subject selected a weight such that they are able to complete 4 repetitions of each task without reaching fatigue. The exercise task saw the subject perform slow controlled concentric contractions followed by eccentric lowering of the weight. This was considered one repetition. Without pause 3 repetitions were repeated during a trial. The same weight was used for both the original and modified versions of each exercise task. The weight varied from 10 – 40 lbs. All six exercises were performed in order from 1 to 6 (a) on a hard flat bench then repeated in the same order on a Swiss ball (1–6b). Two sets occurred for each exercise task. It should be noted that the curl up exercise on the ground and on the Swiss ball does not control for posture, muscle length or other factors which can influence the myoelectric signal. This exercise was mainly included to give the reader a biologically significant reference for the amount of muscle activity occurring during the exercises. An inference of whether a Swiss ball influences muscle activity during a curl up can not be made due the significant differences in posture. For the other exercises studied a neutral lumbar curve could be maintained through out either condition.

### Description of Exercise Movement

After being instrumented, subjects performed the normalization tasks and then the 12 movement tasks. The subjects were instructed to perform the concentric phase for 2 seconds and the eccentric phase for 4 seconds (under the count and supervision of two examiners). Data was collected for 25 seconds during each exercise.

### EMG Processing

The raw myoelectric signal of all trials for both the MVCs and the exercise tasks was processed identically. A linear envelop was calculated by first full wave rectifying (the absolute value of each data point) and then smoothing using a 100 ms moving average with a 50 ms overlap. The average activity over the course of the movement was then calculated for each trial and expressed as a percentage of the activity found during the MVC for each specific muscle and participant.

### Statistical Analysis

A repeated measures ANOVA was then used to determine if a difference in type of supporting surface influenced trunk muscle activity for each muscle. A post hoc Tukey test was used to examine if statistically significant (p < .05) differences exist in trunk muscle activity for the different exercises performed.

### Qualitative Analysis

The difference in muscle activity (expressed as a %MVC) was also calculated for each subject (12), muscle (4) and exercise (6) between the two surface conditions for a total of 240 differences in muscle activity. A change greater than 5% MVC was considered significant. The number of occurrences of a significant increase or decrease in muscle activity was recorded.

## Results

There was no significant difference found between performing each of the six exercises on the Swiss ball and the flat bench for any of the four muscle groups investigated. Tables one to six details the average muscle activity for each muscle group during the six different exercises studied. Twenty six muscles showed significant increases in muscle activity and 22 muscles showed a significant decrease in muscle activity across all conditions.

While there was not a statistically significant increase in any muscle studied, the Internal Oblique muscle tended to have the greatest number of increases in muscle activity when on the Swiss ball compared with the bench. The grouped average internal oblique muscle activity difference between the two conditions tended to be greater than 5% MVC during the curl up, bench press and shoulder press. The bench press exercise showed a trend for muscles to increase their myoelectric signal on the Swiss ball (14 occurrences), while there were only two instances when a muscle's activity decreased more than 5% MVC.

Absolute increases of greater than 11% MVC were seen in the internal oblique in three subjects when performing the bench press on a Swiss ball compared with performing the bench press on the more stable bench. One subject's average activity was approximately 3% MVC on the bench and increased to more than 17% MVC when on the Swiss ball. The biceps curl exercise had 6 occurences of trunk muscles increasing their activity on the Swiss ball while the triceps extension had only one incidence of an increase in muscle activity yet seven occurrences where the average muscle activity decreased more than 5% MVC. The lateral raise exercise showed no trends with only 3 instances of activity increases and 3 instances where muscle activity decreased for all muscles studied. While the shoulder press showed a trend of increased internal oblique activity on the Swiss ball (average absolute difference of 6.52% MVC) there was only 2 instances where any trunk muscle increased its activity on the Swiss ball more than 5% MVC and 5 instances of a muscle showing a decrease in activity of more than 5% MVC.

## Discussion

Replacing an exercise bench with a Swiss ball is not a guarantee for increased trunk muscle activation during upper body strength exercises. There does not appear to be a consistent, generalized response to the addition of a Swiss ball. Statistically, there is no difference between conditions, however the study population showed large variability. This suggests that individuals respond differently to unstable surfaces. Health and fitness professionals who advocate the addition of Swiss balls into exercise programs for the upper limbs can not support this change via the argument that the spinal musculature system is stressed to a greater extent (i.e. increased muscle activity) for all individuals. Importantly, this study does not dismiss the use of Swiss balls for exercises designed to train the trunk muscles. Increases in trunk muscle activity have been documented for these exercises [1] but a general increase in trunk muscle activity was not seen for the upper limb strength exercises studied in the current study. The study's findings also suggest that performing upper limb strength exercises on a Swiss ball does not cause excessive compressive loading due to increased trunk muscle co-activation and therefore may be safe for the low back injured. Changes in compressive or shear loading may different due to postural factors but this was not measured in the present study. What needs to be questioned is what benefit exists in performing upper limb strength exercises on a Swiss ball. An injury risk may still be present because Swiss balls are unstable and may increase the risk of falling and subsequent injury. If the justification is to "train the core" (i.e. recruit agonist-antagonist trunk muscles) then this can't be supported by the results of this study. If other justifications are made (increases in balancing ability, recruitment of secondary hip and leg muscles) then this exercise modification may be reasonable. To minimize injury risk, like any exercise program, exercise difficulty progression should be used in incorporating Swiss balls into an exercise program. Participants who wish to use a Swiss ball in their exercise program should learn the basic upper body strength moves on a stable surface first.

Advocating the use of the Swiss ball in exercise or rehabilitation programs may be justified via other benefits. A recent study has documented short term gains in one legged stance following an exercise program of abdominal curl ups and trunk extensions on a Swiss ball [7]. Swiss balls are often more portable and affordable than a traditional weight bench and may therefore increase exercise compliance and adoption. Anecdotally, Swiss ball classes are popular and enjoyable.

Exercise prescription should be goal dependent. If a therapist merely wants variety in an exercise program and increased exercise compliance and enjoyment then the adoption of a Swiss ball appears reasonable but not justified biomechanically. If the aim of a therapist is to rehabilitate or prevent low back injury then sound biomechanically justified or clinically proven rehabilitation protocols should be advocated. Kavcic et al [6] provides biomechanical support for ground based simple exercises (curl up, side bridge, four point kneeling with leg extension) to adequately train the spinal stabilizers while minimizing the compressive/shear penalty and ensuring adequate spinal stability.

This study is limited to the exercises investigated and weights used. For many of the exercises the weight was not near the maximum load the participant could use. The weight levels were chosen based on the rationale that the same low weight is used during "FitBall" classes geared toward a novice exerciser. Challenging each subject with a greater load may influence trunk muscle activity. Future work should address this limitation. Additionally, only surface electromyographic activity was recorded. The muscles studied are considered global stability muscles and may not adequately represent the muscle activation levels in smaller intersegmental spinal muscles. These muscles have a greater proprioceptive function and if the Swiss ball stresses these muscles to a greater extent this may form the basis for an improved balance effect following training. As well, no measures of range of motion occurred. It is possible that different ranges of motion were seen which would alter the myoelectric signal amplitude without a change in force production due to the length tension properties of muscle. Conversely, more advantageous postures would allow greater force production and hence spinal stability without changes in muscle activity. This study is also limited to conclusions regarding the stability of the surface examined. Other labile surfaces (wobble boards) may result in differences in trunk muscle activity recruitment. These results can not be generalized to all unstable surfaces and all strength training exercises.

## Conclusion

A consistent generalized trend was not seen across subjects (subjects did not uniformly increase trunk muscle activity) when replacing an exercise bench with a Swiss ball during upper limb strength exercises. Individual responses were variable. This suggests that participants respond differently to surface stability modifications.

## Competing interests

The author(s) declare that they have no competing interests.

## Authors' contributions

GL: Conception, design, data collection, data analysis, manuscript preparation

TG, JL, PP, ST: design, data collection, manuscript preparation

**Table 1:** Abdominal curl up exercise muscle activation levels (% MVC, standard deviation in brackets), average difference between surfaces and number of participants whose change in activity was greater than 5% MVC during

**Surface**	Muscles studied during abdominal curl up exercise			
	Rectus Abdominis	External Oblique	Internal Oblique	Erector Spinae
Ball	29.0 (33.1)	23.2 (20.6)	32.9 (27.6)	3.2 (3.5)
				
Bench	25.7 (16.6)	21.1 (13.4)	27.1 (16.4)	6.2 (10.6)
				
Difference	3.36(20.6)	2.17 (11.65)	5.82 (18.2)	3 (7.61)
Increase	2	2	4	0
Decrease	2	3	2	1

**Table 2:** Bench Press Exercise muscle activation levels (% MVC, standard deviation in brackets), average difference between surfaces and number of participants whose change in activity was greater than 5% MVC.

**Surface**	Muscles studied during bench press exercise			
	Rectus Abdominis	External Oblique	Internal Oblique	Erector Spinae
Ball	7.4 (6.3)	5.7 (6.8)	13.5 (9.2)	6.06 (5.9)
				
Bench	4.7 (6.2)	3.1 (2.8)	8.2 (6.8)	3.1 (1.6)
				
Difference	2.68 (6.49)	2.52 (4.7)	5.2 (6.26)	2.93 (5.9)
Increase	4	3	4	3
Decrease	2	0	0	0

**Table 3:** Biceps Curl Exercise muscle activation levels (% MVC, standard deviation in brackets), average difference between surfaces and number of participants whose change in activity was greater than 5% MVC.

**Surface**	Muscles studied during biceps curl exercise			
	Rectus Abdominis	External Oblique	Internal Oblique	Erector Spinae
Ball	5.0 (5.8)	3.0 (4.4)	9.0 (8.4)	8.7 (7.4)
				
Bench	4.2 (5.7)	2.2 (1.9)	6.9 (6.0)	6.5 (6.3)
				
Difference	.83 (2.65)	.74 (2.86)	2.14 (5.79)	2.23 (4.67)
Increase	1	1	1	3
Decrease	0	0	0	0

**Table 4:** Lateral raise exercise muscle activation levels (% MVC, standard deviation in brackets) average difference between surfaces and number of participants whose change in activity was greater than 5% MVC.

**Surface**	Muscles studied during lateral raise exercise			
	Rectus Abdominis	External Oblique	Internal Oblique	Erector Spinae
Ball	5.2 (6.2)	3.0 (3.3)	7.8 (7.6)	3.0 (2.0)
				
Bench	5.3 (8.5)	2.0 (1.9)	6.5 (6.1)	4.0 (4.2)
				
Difference	-.07 (3.21)	.99 (2)	1.23 (1.97)	-1 (4.26)
Increase	1	1	1	0
Decrease	2	0	0	1

**Table 5:** Shoulder press exercise muscle activation levels (% MVC, standard deviation in brackets) average difference between surfaces and number of participants whose change in activity was greater than 5% MVC.

**Surface**	Muscles studied during shoulder press exercise			
	Rectus Abdominis	External Oblique	Internal Oblique	Erector Spinae
Ball	6.01 (6.29)	4.1 (5.4)	21.7 (31.5)	3.7 (3.3)
				
Bench	6.9 (9.6)	3.5 (3.6)	15.2 (15.2)	13.4 (30.3)
				
Difference	-.98 (4.29)	.6 (2.59)	6.52 (30.23)	-1.07 (4.62
Increase	0	1	1	0
Decrease	1	0	3	1

**Table 6:** Triceps extension exercise muscle activation levels (% MVC, standard deviation in brackets) average difference between surfaces and number of participants whose change in activity was greater than 5% MVC.

Surface	Muscles Studied during triceps extension exercise			
	Rectus Abdominis	External Oblique	Internal Oblique	Erector Spinae
Ball	4.3 (3.6)	3.7 (4.3)	13.5 (12.5)	3.4 (3.3)
				
Bench	9.8 (16.6)	4.3 (4.0)	16.3 (16.5)	3.1 (2.0)
				
Difference	-5.51 (14.04)	.67 (1.91)	2.76 (5.8)	.31 (3.42)
Increase	0	0	0	1
Decrease	3	1	3	0
